# Left Ventricular Wall Thickness in Hypertrophic Cardiomyopathy and Cardiac Amyloidosis: A Multimodality Imaging Comparison

**DOI:** 10.3390/jcm15208083

**Published:** 2026-10-18

**Authors:** Zubair Bashir, Syed Bukhari, Michelle Yang, Huda Jamil, Mohamed Bilal Abdallah, Esosa G. Odigie-Okon, Wissam Khalife

**Affiliations:** 1 Department of Cardiology, University of Texas Medical Branch, Galveston, TX 77755, USA; 2 Division of Cardiology, Johns Hopkins University School of Medicine, Baltimore, MD 21205, USA; 3 Allama Iqbal Medical College, Lahore 54700, Pakistan; 4 Department of Health and Human Performance, University of Houston, Houston, TX 77204, USA

**Keywords:** left ventricular hypertrophy, hypertrophic cardiomyopathy, cardiac amyloidosis, echocardiography, cardiac magnetic resonance imaging

## Abstract

Hypertrophic cardiomyopathy (HCM) and cardiac amyloidosis (CA) are key differential diagnoses in patients presenting with an increase in left ventricular wall thickness. Despite advances in multimodal imaging, both conditions remain underrecognized due to overlapping morphological and clinical features that complicate diagnosis and treatment. However, their distinct pathophysiological mechanisms—myocardial hypertrophy and myofibrillar disarray in HCM versus extracellular deposition of misfolded amyloid protein in CA—have direct implications for management. Timely and accurate diagnosis is critical to optimizing patient care and preventing serious adverse outcomes, including sudden cardiac death. Epidemiological factors such as age, sex, and geographic distribution, along with specific clinical characteristics, can increase pretest probability and guide targeted diagnostic evaluation. As we enter a new era of precision medicine, this review comprehensively discusses the epidemiological, clinical, and imaging distinctions between HCM and CA to enhance diagnostic accuracy and clinician awareness to provide optimal care delivery.

## 1. Introduction

**Figure 1:**
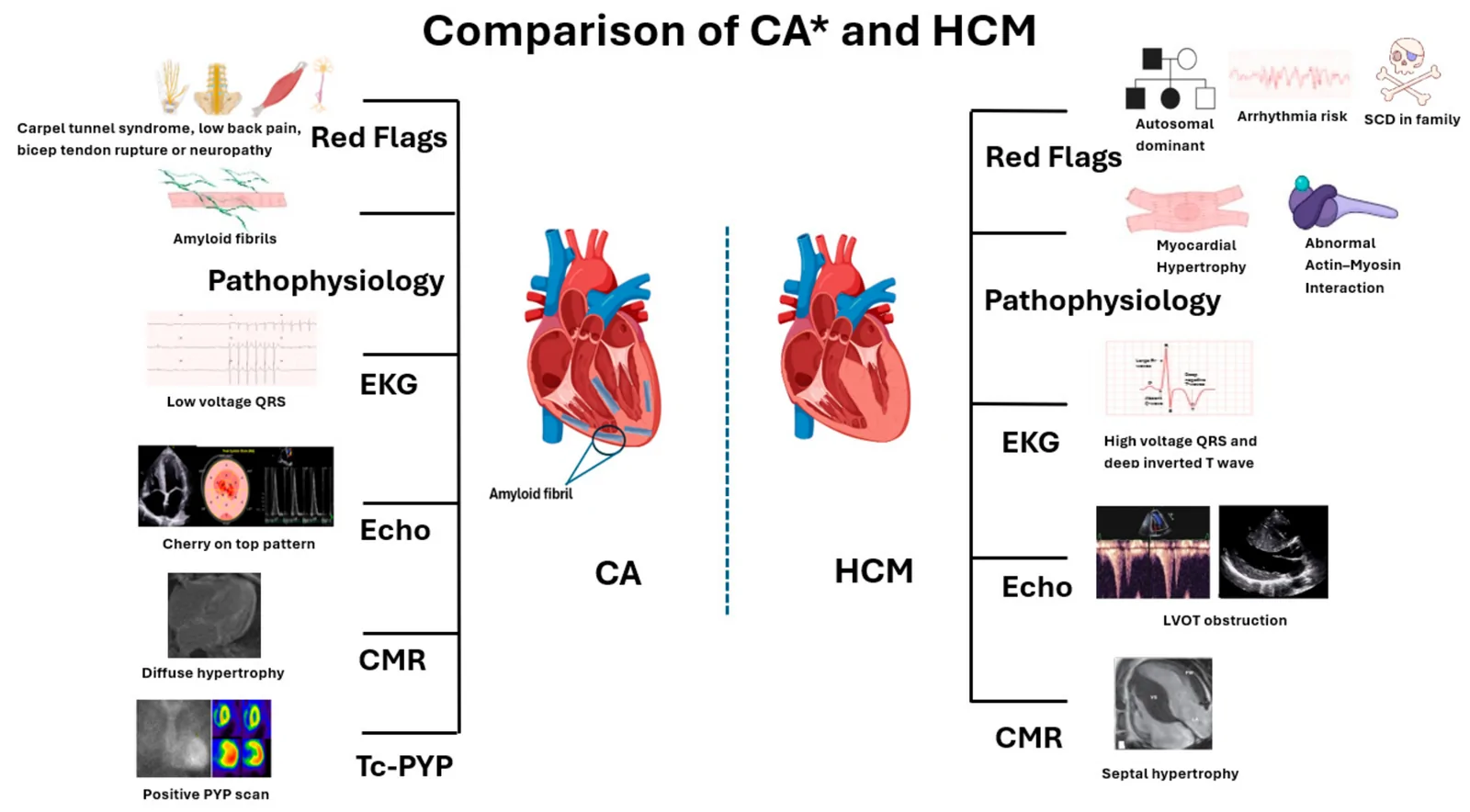
Central illustration. * Transthyretin cardiac amyloidosis.

The cardinal feature of hypertrophic cardiomyopathy (HCM) and cardiac amyloidosis (CA)—two distinct entities—is increased myocardial wall thickness. They share numerous characteristics in addition to an increase in myocardial wall thickness, which include atrial dilatation, valvular thickening, symptoms of heart failure, and heightened risks for arrhythmia. Each entity can mimic the clinical manifestations of the other, posing a diagnostic challenge for clinicians. In addition, both entities carry increased morbidity and mortality rates if they remain undiagnosed. Despite increased awareness and improved diagnostic capabilities, particularly with advanced imaging modalities, CA and HCM remain underrecognized. This is particularly true among asymptomatic individuals or those with subtle phenotypic features. While detection among older adults has improved due to increased imaging use and survivorship, many cases remain clinically silent. Therefore, assessing the common and distinguishing features between the two entities is imperative to ensure timely diagnosis and effective management. We surveyed the contemporary literature up to March 2026, focusing on hypertrophic cardiomyopathy, cardiac amyloidosis, and increased left ventricular wall thickness across multimodality imaging platforms—including echocardiography, cardiac magnetic resonance imaging, nuclear scintigraphy, and strain imaging—to provide a comprehensive review of the pretest probability and the role of multimodal imaging in the timely diagnosis of both underdiagnosed entities (Figure 1).

## 2. Epidemiology

### 2.1. Hypertrophic Cardiomyopathy

HCM is a common inherited cardiomyopathy, with an estimated global prevalence between 0.2% and 0.5% (1 in 500 to 1 in 200 individuals) [1]. The worldwide incidence is approximately 4.7 per 100,000 individuals annually [2]. Interestingly, two seminal epidemiologic studies of HCM that used imaging criteria to define HCM—the UK Biobank and CARDIA—reported a similar HCM prevalence of ~0.2% (1 in 500) despite using different imaging modalities to diagnose HCM, involving participants from different geographic regions, and the studies being conducted at distinct time periods [3,4]. Notably, the UK Biobank enrolled predominantly older adults with a mean age of 65 years, while the CARDIA study focused on younger adults aged 18–30 years, underscoring consistent disease detection across age groups.

However, real-world data from administrative claims, using International Classification of Diseases, Ninth Revision (ICD-9) diagnostic codes, reported a treated prevalence of just 1 in 3000 individuals in the U.S in 2013. Most of the diagnoses were reported in midlife and only 43% of cases were reported in women [5]. These findings highlight the persistent under-recognition of HCM in clinical practice. However, the prevalence of HCM rose from 19.7 to 81.5 cases per 100,000 individuals between 1985 and 2016, and the mean age at diagnosis increased to 61.2 years. Moreover, the male-to-female diagnostic ratio narrowed from 2:1 to 1.5:1 [6,7]. Despite improvement in identifying HCM over the last few decades, it remains underrecognized, and a comprehensive framework is much needed to assist clinicians in disease identification and establishing a diagnosis.

A clinically significant phenotype, obstructive hypertrophic cardiomyopathy (oHCM), characterized by dynamic left ventricular outflow tract obstruction (LVOTO), which is attributed to causing most of the symptoms, has demonstrated a relatively stable prevalence rate of ~1.6 per 10,000 individuals between 2016 and 2018 based on the administrative claims MarketScan database [8].

### 2.2. Cardiac Amyloidosis

The prevalence of CA differs based on the type of variant. It is generally divided into hereditary transthyretin amyloidosis (ATTRv), wild-type transthyretin amyloidosis (ATTRwt), and immunoglobulin light chain amyloidosis (AL).

- ATTRwt: It is a predominant form of amyloidosis among the elderly, particularly in males and those of Caucasian ancestry. Over recent decades, the proportion of diagnosed CA cases attributed to ATTRwt has increased substantially, from less than 3% between 1987 and 2009 to approximately 25% between 2016 and 2019 [9]. This increase likely reflects, at least in part, greater disease awareness and recognition, advances in noninvasive diagnostic techniques, and increased detection of ATTR rather than a true increase in population incidence [10]. More than 90% of patients with ATTRwt exhibit cardiac involvement, with a median age at diagnosis of 75 years. Following diagnosis, the reported median survival is 3.6 years [11].
- ATTRv: ATTRv is caused by more than 120 distinct mutations in the TTR gene, inherited in an autosomal dominant manner. The disease varies in terms of onset, geographic distribution, primary phenotype, and progression, all of which are influenced by the specific pathogenic variant and fibril composition [12]. The exact prevalence of ATTRv remains unclear. In the United States, the most frequently observed pathogenic variant is the substitution of valine with isoleucine at position 122 (V122I), which is found in 3–4% of African Americans [13]. Phenotypically, V122I-ATTRv closely resembles ATTRwt, predominantly affecting men and leading to late-onset cardiomyopathy with minimal neurological symptoms, typically emerging around the age of 70.
  The second-most-common U.S. pathogenic variant, T60A, is linked to Northern Irish ancestry and presents as a mixed cardiomyopathy–neuropathy syndrome [14]. On a global scale, the V30M pathogenic variant is the most frequently encountered, manifesting in both early- and late-onset disease [15]. In endemic regions such as Sweden, Portugal, and Japan, early-onset disease, which appears before age 50, is primarily characterized by neurological symptoms. Notably, Swedish patients with ATTR V30M tend to develop symptoms later compared to individuals from Japan and Portugal [16]. In contrast, the late-onset variant often emerges in non-endemic areas, frequently presenting with cardiac symptoms alongside severe neuropathy [17,18]. Certain pathogenic variants are more prevalent in specific regions, such as L111M in Denmark and I68L in Italy [19].
- AL: AL remains an important cause of cardiac amyloidosis, although its relative frequency varies according to the population and referral setting [9]. Each year, around 2200 new cases of AL are diagnosed in the United States, with a median age of onset of 65 years and a higher prevalence in males than in females. Compared to ATTR, AL progresses more aggressively due to the direct cardiotoxic effects of amyloidogenic light chains [20]. AL can involve multiple organs, most commonly the heart, kidneys, liver, gastrointestinal tract, peripheral and autonomic nervous systems, and soft tissues, whereas central nervous system involvement is uncommon. Among these, cardiac involvement is a key determinant of prognosis. Patients with untreated AL and heart failure have a median survival of six months if left untreated [21].

## 3. Pathophysiology

### 3.1. Hypertrophic Cardiomyopathy

**Figure 2:**
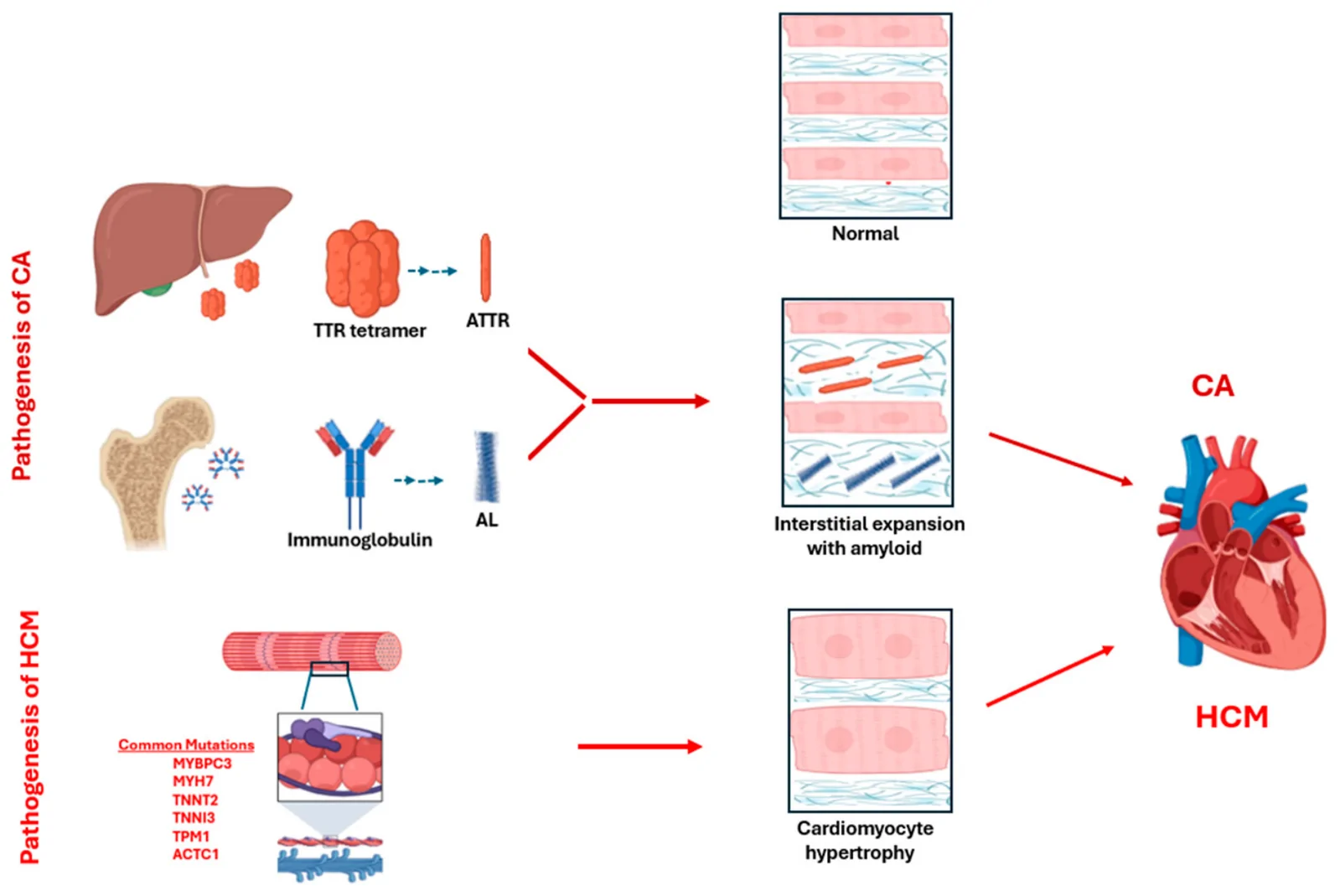
Pathogenesis of HCM and CA. Both HCM and CA present with an increase in the LV thickness, but due to different pathogenetic mechanisms.

HCM is an autosomal dominant disorder of the myocardial sarcomere with variable penetrance [22]. The disease is primarily driven by pathogenic variants in genes encoding sarcomere proteins, resulting in abnormal myocyte architecture characterized by hypertrophy, myofibrillar disarray, interstitial fibrosis, and impaired myocardial contractility (Figure 2).

The clinical expression of HCM is highly heterogeneous. Variability in age of onset, symptom burden, arrhythmia risk, progression to heart failure, susceptibility to SCD, and the pattern and severity of myocardial hypertrophy is largely attributed to genotype–phenotype interactions [23]. However, multiple pathophysiological mechanisms have been proposed to explain this complexity, including abnormal myocardial bioenergetics. However, the underlying mechanism is the disruption of the actin–myosin interaction, leading to ventricular stiffness, diastolic dysfunction, asymmetric myocardial hypertrophy, ischemia, and conduction system abnormalities [24,25].

While over eight genes have been implicated in sarcomere dysfunction, the majority of familial HCM cases are due to mutations in either the myosin heavy chain 7 (MYH7) or myosin-binding protein C (MYBPC3) genes, together accounting for roughly 50% of genetically diagnosed cases [26,27]. Less commonly, mutations in genes encoding troponin T (TNNT2), troponin I (TNNI3), and alpha-tropomyosin (TPM1) are observed, making up fewer than 10% of cases [27,28]. Despite these genetic associations, a considerable percentage of patients with clinically diagnosed HCM do not exhibit detectable sarcomere mutations, as shown by Ingles et al., who did not find any identifiable mutation in 40% of HCM patients. Interestingly, these patients without genetic susceptibility had late-onset disease and milder phenotypes [29].

### 3.2. Cardiac Amyloidosis

CA is characterized by the deposition of misfolded proteins as insoluble amyloid fibrils within the myocardium, disrupting normal cardiac architecture and function [30]. These deposits lead to myocardial stiffening, which impairs both diastolic and, eventually, systolic function, and contributes to the development of heart failure and arrhythmias [31]. More than 90% of CA cases are attributed to amyloid fibrils derived from either ATTR or AL. ATTR occurs due to the destabilization of the transthyretin tetramer, resulting in monomers that misfold and aggregate into amyloid fibrils [32]. This form can be classified into two subtypes: ATTRwt and ATTRv. In contrast, ATTRv is caused by an inherited pathogenic variant in the transthyretin gene. Clinical manifestations vary based on the specific pathogenic variant. The V122I variant, prevalent in the U.S., particularly affects older African American men and is primarily associated with cardiac involvement [13] (Figure 2).

AL, on the other hand, is plasma cell dyscrasia resulting from the overproduction of misfolded immunoglobulin light chains by clonal plasma cells in the bone marrow [33]. These circulating light chains misfold and deposit as amyloid fibrils, typically affecting multiple organs. While the kidneys are frequently involved, cardiac involvement is the most significant predictor of prognosis. AL amyloidosis tends to follow a more aggressive clinical course than ATTR, with a median survival of just six months following the onset of cardiac symptoms if left untreated [34].

## 4. Clinical Characteristics

### 4.1. Hypertrophic Cardiomyopathy

HCM primarily affects the heart and presents as an unexplained increase in LV wall thickness in the absence of loading conditions and relevant phenocopies. It is characterized by extensive clinical heterogeneity. Patients may present with a wide range of symptoms, including palpitations, syncope, heart failure symptoms, angina, and even SCD. However, there is a phenotypic subset of patients that remain asymptomatic and are identified incidentally. The predominant pathophysiologic mechanisms underlying symptoms include abnormal hypertrophy of the LV wall, abnormalities of valvular and sub-valvular apparatus causing LVOTO, and abnormal energetics in the absence of secondary causes, such as longstanding hypertension or aortic stenosis [22]. Based on the phenotypic characteristics, HCM patients are classified into three groups: (1) those without LVOTO, (2) those with latent LVOTO, unmasked only on provocative testing or exercise, and (3) those with LVOTO at rest. The combination of LVOTO and mitral valve (MV) abnormalities, such as systolic anterior motion (SAM) of the anterior leaflet of MV, can compromise cardiac output and cause syncopal episodes. When syncopal episodes occur in a patient with a family history of syncope or SCD and in the absence of an identifiable cause such as aortic stenosis, HCM should be suspected and further diagnostic evaluation should be performed.

HCM also increases arrhythmogenicity, and atrial fibrillation is a common arrhythmia in HCM that may present as palpitations or even with recurrent syncopal episodes. Moreover, dyspnea on exertion in the absence of commonly known risk factors such as hypertension, diabetes, obesity, aortic stenosis, or smoking, along with increased LV wall thickness, should raise suspicion of HCM [22,35].

Patients with HCM may also present with anginal chest pain, posing a diagnostic dilemma. In the absence of significant epicardial CAD, the presence of markedly thickened LV walls on echocardiography should prompt consideration of HCM. This ischemic-like discomfort arises from a mismatch between myocardial oxygen supply and demand, driven by hypercontractility of hypertrophied myocardium, elevated intracavitary pressures, microvascular dysfunction, and reduced coronary flow reserve [36].

**Figure 3:**
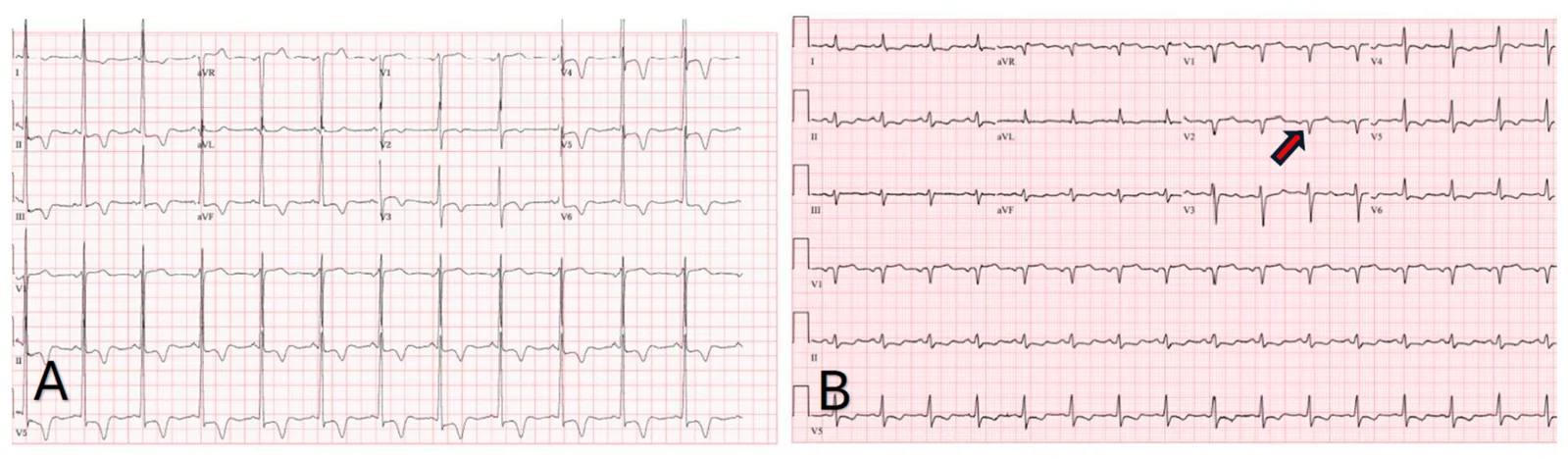
(**A**) EKG of HCM patient shows left ventricular hypertrophy and deep inverted T waves in inferolateral leads consistent with the Yamaguchi pattern of apical HCM. (**B**) EKG of a CA patient shows diffuse low-voltage QRS complexes and a pseudo-infarct pattern (red arrow).

Electrocardiographic (EKG) findings in HCM are variable but frequently show signs of LV hypertrophy, such as high-voltage QRS complexes. Other common features include deep T-wave inversions in the precordial, high lateral, and inferior leads, and deep, narrow Q waves in the lateral and high lateral leads. In more advanced stages, P mitrale may be seen, indicative of left atrial (LA) enlargement secondary to chronic elevation in filling pressures due to diastolic dysfunction and mitral regurgitation [37] (Figure 3). Although these EKG abnormalities are nonspecific, in the appropriate clinical setting (absence of risk factors for conventional coronary artery disease (CAD) or secondary causes of LV hypertrophy), they may provide valuable diagnostic clues.

### 4.2. Cardiac Amyloidosis

The initial assessment of CA relies heavily on clinical evaluation, beginning with a thorough history and EKG findings. Recognizing extracardiac manifestations can be especially helpful in differentiating CA from other cardiac conditions. Common systemic clues include bilateral carpal tunnel syndrome, lumbar spinal stenosis, prior orthopedic joint replacements (hip, knee, or shoulder), proteinuria, and signs of autonomic neuropathy such as orthostatic hypotension. Among these, carpal tunnel syndrome is often one of the earliest manifestations—frequently preceding cardiac involvement by 5 to 10 years—making it a critical diagnostic clue [38].

On EKG, low-voltage QRS complexes—attributable to amyloid infiltration disrupting electrical conduction—are found in approximately 35% of ATTR and 55% of AL cases, though not universally present [38,39]. The coexistence of low voltage with increased LV wall thickness is a classic yet paradoxical finding that should heighten suspicion for CA. Additional EKG abnormalities commonly seen include atrial fibrillation, pseudo-infarct patterns, and conduction abnormalities such as first-degree atrioventricular (AV) block [40]. Atrial fibrillation is particularly frequent, likely resulting from elevated filling pressures and direct amyloid-induced atrial myopathy [41,42]. Ventricular tachyarrhythmias (VT) and bradyarrhythmias may also occur in some patients [43,44] (Figure 3).

Laboratory evaluation often reveals persistent elevations in high-sensitivity troponin, which carries prognostic implications [45]. Myocardial injury in CA stems from both amyloid-related mechanisms—including precursor protein toxicity, interstitial infiltration, and vascular involvement—and non-amyloid contributors such as systolic and diastolic dysfunction, heart failure, underlying coronary artery disease, tachyarrhythmias, and hypotension [45].

## 5. Echocardiography Features

### 5.1. Hypertrophic Cardiomyopathy

Echocardiography is pivotal in diagnosing HCM, differentiating it from phenocopies that present with increased LV wall thickness. It also plays a central role in defining the HCM phenotype—whether obstructive or nonobstructive—and contributes to the risk stratification for SCD. The diagnosis of HCM is established by identifying a maximum LV wall thickness of ≥15 mm in the absence of any loading conditions that would cause such disproportionate LV hypertrophy (LVH). The diagnosis can also be established with LV wall thickness of ≥13 or 14 mm in the presence of positive genetic testing for pathogenic or likely pathogenic variants, or a family history of HCM. Asymmetric hypertrophy is more common than symmetric hypertrophy and can involve different segments of the myocardium with or without obstruction. Hypertrophy of the basal segment is most common; however, other hypertrophic patterns such as sigmoidal, midventricular, apical, septal curvature, and neutral septum are also seen. A common echocardiographic feature that raises suspicion for HCM is asymmetric septal hypertrophy, with a septal-to-posterior wall thickness ratio of 1.5:1. Small LV cavity size and hypercontractile LV wall motion are also commonly seen due to the disproportionate LV wall thickness [22].

In addition to LV hypertrophy, diastolic dysfunction, systolic dysfunction in advanced stages, and morphological pathologies of the MV apparatus are commonly seen. Common MV abnormalities include increased leaflet length, thickening and distorted leaflet coaptation, anomalous papillary muscle insertion, and anteriorly displaced papillary muscles. SAM of the MV may cause LVOTO and mitral regurgitation in HCM patients. Although SAM is not pathognomonic for HCM, it is highly indicative of HCM with a specificity of 98% [46]. Groarke et al. reported that among 192 genotyped patients, 79 patients with subclinical HCM demonstrated elongated anterior MV leaflets and anteriorly displaced papillary muscles compared to normal healthy subjects. These findings were also observed in 50 patients with overt HCM. This shows that MV apparatus abnormalities are present not just in patients with clinically overt HCM, but also in silent carriers of the disease. Furthermore, structural pathologies of the MV may precede LVH as part of the early phenotypic expression of HCM sarcomere mutations [47].

Doppler interrogation across the LVOT helps to rule out obstructive phenotype, which determines symptomatology and also helps in prognostication and therapy selection. Doppler echocardiography in dynamic obstruction shows a characteristic signal with a late-peaking dagger-shaped appearance [48]. Moreover, in some cases of severe obstruction, a characteristic “lobster claw” Doppler tracing abnormality is observed due to midsystolic decrease in LV ejection velocity [49,50]. However, LVOTO is a dynamic phenomenon influenced by heart rate, blood pressure, volume status, and alterations in preload and afterload; therefore, LVOTO is not necessarily observed during rest. Therefore, in patients with high clinical suspicion and negative resting gradients, provocative testing with Valsalva or changing position from squatting to standing should be performed. In addition, exercise stress echocardiography is recommended for symptomatic patients with a gradient < 50 mmHg. Stress echocardiography has additional advantages for assessing exercise capacity and any inducible ischemia in addition to evaluating LVOT gradient. oHCM is diagnosed with a LVOT gradient of ≥30 mmHg, whether at rest or with provocative testing, including exercise stress echocardiography. However, a gradient of ≥50 mmHg increases the likelihood of symptoms and is important for therapy selection. In addition, LVOT gradient as a continuous variable is also a component of a risk stratification tool that estimates the SCD risk and guides decisions regarding defibrillator therapy [22]. Higher gradient is associated with a higher risk of SCD.

Furthermore, LA enlargement in the presence of unexplained asymmetric LV wall hypertrophy provides prognostic information. Increased LA diameter of > 48 mm elevates risk for atrial arrhythmias. LA size as a continuous variable is also a component of the risk calculator assessing the probability of SCD and contributing to the decision-making for ICD implantation for primary prevention. LA enlargement is likely a sequela of long-standing diastolic dysfunction and mitral regurgitation due to LVH.

**Figure 4:**
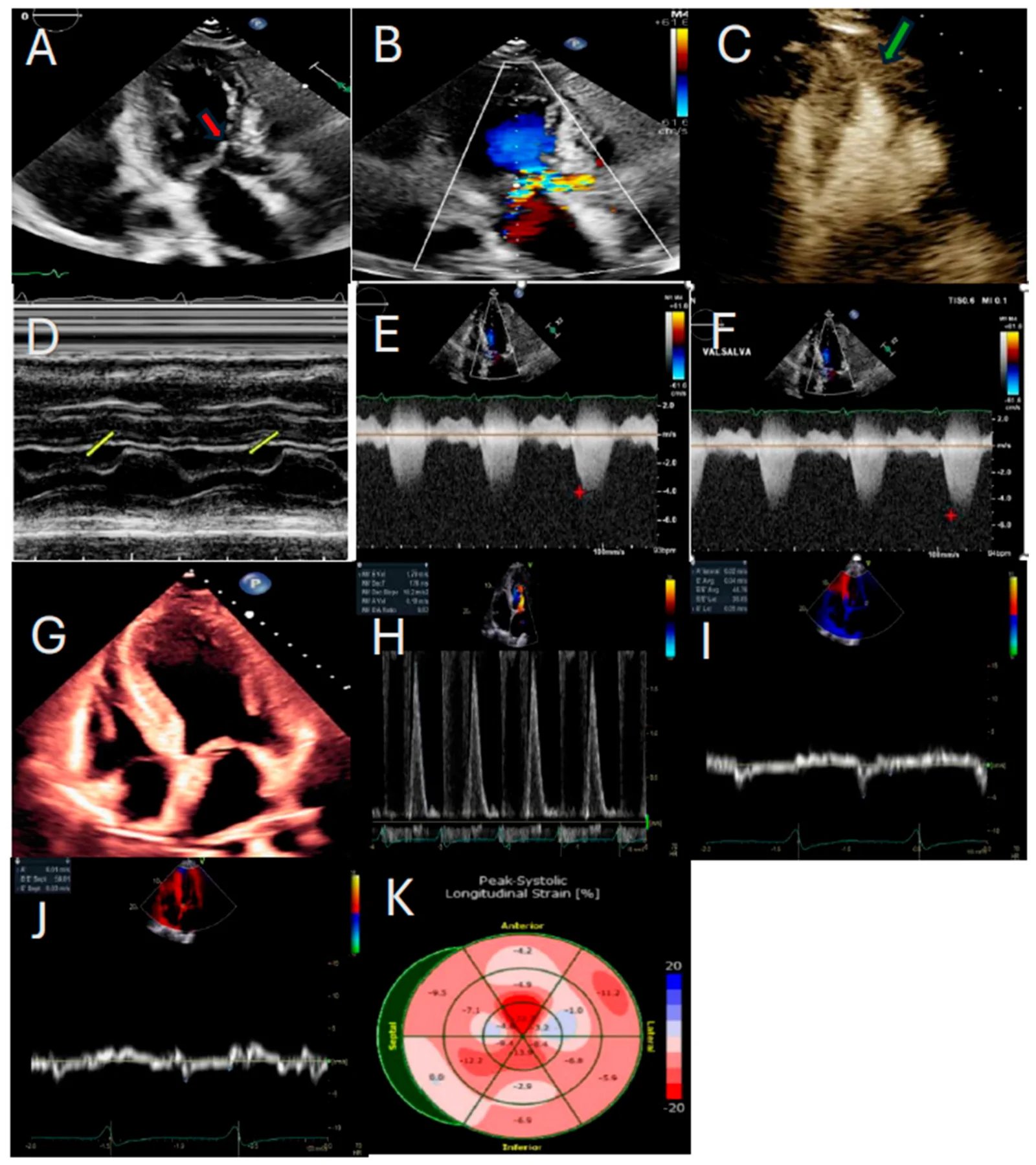
The upper two rows show echocardiographic features of HCM. Three-chamber view showing asymmetric severe septal hypertrophy with left ventricular outflow tract obstruction from systolic anterior motion of the mitral valve (red arrow) (**A**), resulting in posteriorly directed mitral regurgitation on color Doppler (**B**); note turbulence in the left ventricular outflow tract. The four-chamber view shows apical hypertrophy of the left ventricular wall (green arrow)- a variant of HCM (**C**). M-Mode shows late systolic left ventricular outflow tract obstruction from the anterior mitral leaflet (yellow arrows) (**D**). Continuous-wave Doppler through the left ventricular outflow tract shows a significant resting outflow tract gradient (64 mmHg) (**E**), which increases to 100 mmHg with Valsalva maneuver (red star) (**F**). The bottom two rows show echocardiographic features of CA. The four-chamber view shows granular sparkling appearance of myocardium (**G**). High E, low A, and E/A > 2 (**H**), lateral and septal wall tissue Doppler with reduced e′ and a′ velocities (**I**,**J**) consistent with a restrictive pattern. Reduced global longitudinal strain with apical sparing, “cherry on top” pattern on Speckle tracking echocardiography (**K**).

Lastly, speckle tracking echocardiography (STE) can assist with more accurate quantification of systolic function in HCM. Despite underlying myocardial dysfunction and fibrosis, traditional echocardiography parameters are unable to quantify LV systolic function accurately, likely due to small LV cavity size and disproportionate increase in the LV wall thickness. STE reporting global and regional longitudinal strain and strain rate can identify subtle myocardial dysfunction. An absolute global longitudinal strain (GLS) of less than 10% is associated with myocardial fibrosis and a higher risk of adverse events such as ventricular arrhythmias [51]. Moreover, different patterns of regional segmental strain variability are seen based on the involvement of LV segments [52] (Figure 4) (Table 1).

### 5.2. Cardiac Amyloidosis

Echocardiography serves as a cornerstone in the evaluation of CA, offering high sensitivity for detecting both structural and functional myocardial abnormalities [53]. Amyloid infiltration leads to characteristic features such as concentric thickening of the ventricular walls, diastolic dysfunction with restrictive physiology, and elevated filling pressures—findings that help distinguish CA from conditions like severe aortic stenosis or poorly controlled hypertension [53]. One echocardiographic feature that should raise suspicion for CA is increased septal wall thickness (>12 mm) in the absence of loading conditions. Previously, a distinctive granular or “speckled” appearance of myocardium was commonly seen due to amyloid deposits. However, the use of harmonic imaging has reduced the reliability of this finding [53,54].

Tissue Doppler imaging typically reveals a restrictive filling pattern, with significantly reduced myocardial velocities (e′, a′, and s′), often measuring less than 5 cm/s—reflecting impaired longitudinal function [53]. Pericardial effusion, another common finding in CA, may also be present and should raise clinical suspicion [53].

Biatrial enlargement is frequently observed and reflects chronically elevated filling pressures. Strain imaging of the LA often reveals global impairment across all phases—reservoir, conduit, and contractile function [55,56]. Additionally, an interatrial septal thickness exceeding 6 mm has been reported as a highly specific echocardiographic marker for CA, especially when taken along with other echocardiographic and clinical features of CA [57].

Other suggestive features include unexplained increase in the thickness of the right ventricular (RV) free wall and dilation of right-sided cardiac chambers, both of which may indicate RV involvement by amyloid infiltration [57]. STE provides further diagnostic value by demonstrating markedly reduced GLS, especially in the basal and mid-ventricular segments, with relative preservation of apical function. This apical sparing pattern produces the classic “cherry-on-top” appearance on the GLS bull’s-eye plot—considered a hallmark finding in CA [58]. A recent systematic review and meta-analysis comprising 37 studies and ~2800 patients with CA reported a pooled sensitivity of 61% (95% CI: 54–68%) and pooled specificity of 83% (95% CI: 80–86%) for the apical sparing pattern in detecting CA [59]. However, this finding is not exclusive to CA. The apical sparing strain pattern is not uncommon in patients with end-stage kidney disease (ESKD), and studies have demonstrated that it cannot be reliably used as a tool to suspect CA in this cohort [60]. Similarly, relative apical longitudinal strain preservation is observed in approximately one-fifth of patients with severe aortic stenosis without CA [61]. Hence, the presence of apical sparing pattern should be interpreted with caution and always within the broader clinical context, especially in patients with ESKD and severe aortic stenosis (Figure 4) (Table 1).

**Table 1:** Comparison of echocardiography findings between HCM and CA.

Feature	HCM	CA
**LV Wall Thickness**	≥15 mm (diagnostic) ≥13–14 mm with genetic/family history	>12 mm, often concentric thickening
**Hypertrophy Pattern**	Asymmetric (common) Segmental involvement	Concentric thickening
**Cavity Size**	Small LV cavity	May be normal or small
**Systolic Function**	Hypercontractile LV wall motion (early); later stages may show LV systolic dysfunction	Early, preserved EF but impaired GLS. Reduced EF in advanced disease
**Diastolic Function**	Diastolic dysfunction due to stiff hypertrophied LV	Severe restrictive filling pattern with elevated filling pressures
**Mitral Valve Abnormalities**	Elongated/thickened leaflets Anteriorly and apically displaced papillary muscles SAM and MV regurgitation	MV regurgitation may occur; structural abnormalities less prominent
**LVOT Obstruction**	Dynamic obstruction with SAM seen in 2/3rd of patients Dagger-shaped Doppler signal “Lobster claw” pattern; gradient ≥ 30 mmHg (obstructive); ≥50 mmHg-part of therapy selection Gradient is part of SCD risk calculator	LVOTO is not commonly observed
**Atrium**	LA enlarged in 1/3rd of patients, can be a predictor of AF LA size is a component of SCD risk calculator	Biatrial enlargement All phases of LA strain are impaired
**Right Ventricle Involvement**	Less common	Increase in RV wall thickness
**Tissue Doppler Findings**	Variable, may show reduced e′ in late stages	Restrictive pattern; reduced e′, a′, s′ < 5 cm/s
**Speckle Tracking Echocardiography**	Absolute GLS < 10% has prognostic significance Segmental variability by hypertrophy location	GLS markedly reduced in basal/mid segments with preserved apical strain—“cherry-on-top” bull’s-eye pattern in ~2/3rd of patients

HCM: hypertrophic cardiomyopathy, CA: cardiac amyloidosis, AF: atrial fibrillation, SCD: sudden cardiac death, LVOTO: left ventricular outflow tract obstruction, LV: left ventricle, EF: ejection fraction, MV: mitral valve, SAM: systolic anterior motion, GLS: global longitudinal strain.

## 6. CMR Features

### 6.1. Hypertrophic Cardiomyopathy

Cardiac magnetic resonance (CMR) imaging provides detailed assessment of cardiac structure and function without any limitations of acoustic windows [22,62,63]. CMR is particularly valuable in cases of unexplained LVH, risk stratification for SCD, and planning for septal reduction therapy. It allows for the accurate visualization of wall motion abnormalities and turbulent flow jets associated with LVOTO [64]. Additionally, it can identify abnormal papillary muscle morphology, including hypertrophy, anomalous insertion, or direct MV attachment—findings that are important when considering septal reduction, as alcohol septal ablation may be less effective in these cases, often favoring surgical myectomy instead [65].

**Figure 5:**
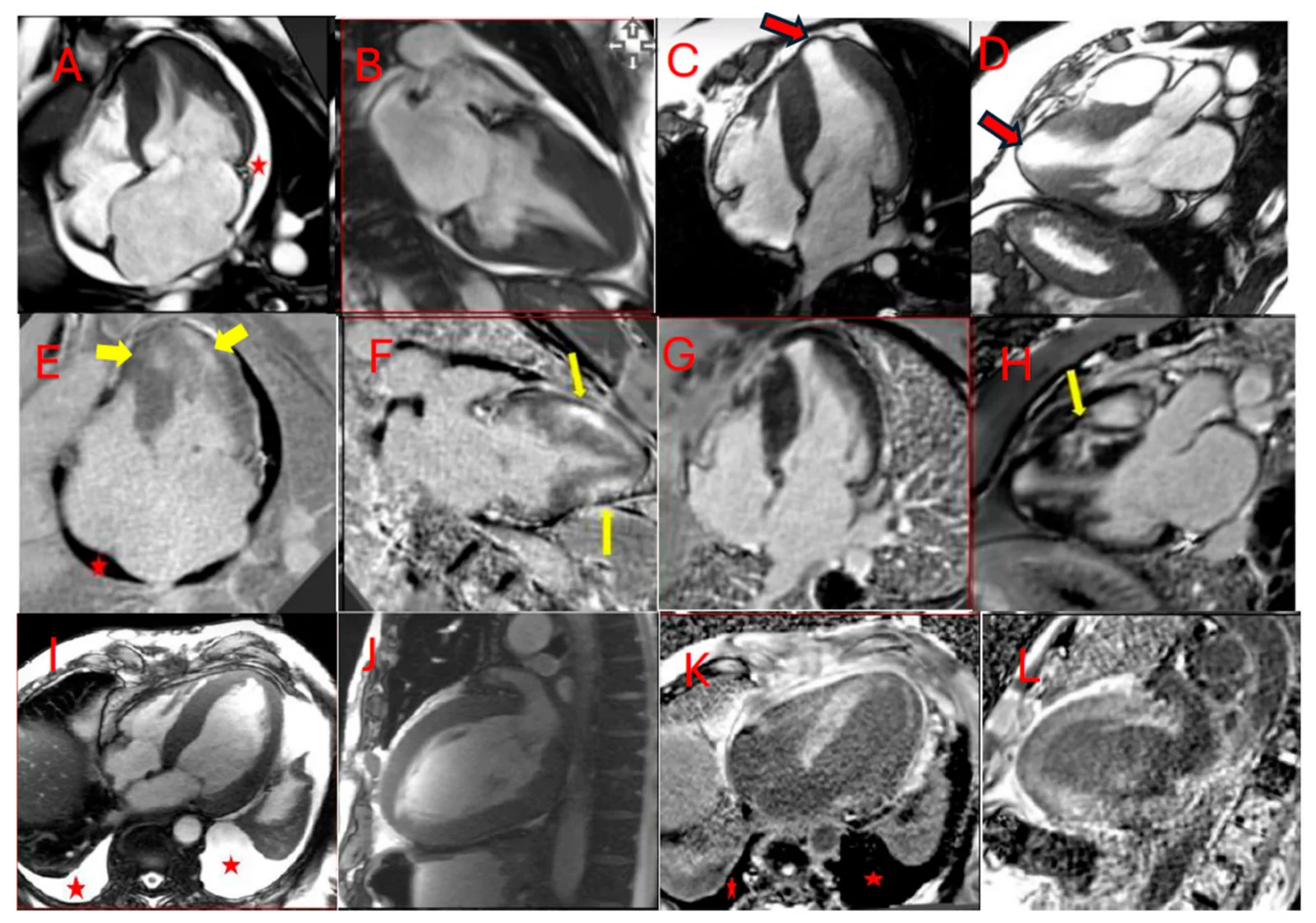
The upper two rows show CMR features of apical HCM. Cine images in four-chamber (**A**) and two-chamber (**B**) views showing apical HCM with varying degrees of hypertrophy and small pericardial effusion (asterisk). Cine images in four-chamber (**C**) and three-chamber (**D**) views with varying degrees and areas of hypertrophy showing concentric HCM with apical aneurysm (red arrow). The middle row (**E**,**F**,**G**,**H**) shows corresponding delayed enhancement images with patchy mid-wall fibrosis in the mid-to-apical segments in apical HCM and basal-to-mid anterior and anteroseptal walls in apical HCM (yellow arrows). The bottom row shows CMR features of CA. Severe concentric hypertrophy (**I**,**J**) in four-chamber and two-chamber views, with thickened valves and the interatrial septum. Corresponding figures (**K**,**L**) show diffuse subendocardial delayed enhancement, including the atria, consistent with extracellular space expansion and/or fibrosis. Large left and small right bilateral pleural effusions are present (asterisk), consistent with heart failure.

A major strength of CMR is its ability to detect myocardial fibrosis or the expansion of extracellular space using late gadolinium enhancement (LGE). In HCM, LGE typically localizes to segments of maximum LV wall hypertrophy and usually does not correspond to the coronary artery distribution. It commonly appears as isolated or multiple patchy LGE at the mid-ventricular wall or along the right ventricular insertion points on the interventricular septum. However, it may also be diffuse, focal, or involve the papillary muscles [66,67,68]. LGE patterns are also critical for differentiating HCM from its various phenocopies, including infiltrative cardiomyopathies and storage disorders, which may mimic LVH [68,69] (Figure 5). LGE has significant prognostic implications and is associated with ventricular arrhythmias and SCD. Chan et al., in an observational study, demonstrated (N = 1293) that the extent of LGE correlates with an increased risk of SCD (aHR: 1.46 per 10% increase in LGE, *p* = 0.002) in HCM patients. Those with LGE ≥ 15% of LV mass had a 2-fold increase in SCD risk, even when they were otherwise considered low-risk [70]. These findings underscore the emerging role of LGE in risk stratification.

Native T1 mapping identifies myocardial fibrosis and interstitial expansion, even in LGE-negative HCM patients. Increased T1 values correlate with disease severity and wall thickness [71]. Extracellular volume (ECV) fraction differentiates HCM from athlete’s heart and correlates with fibrosis burden and adverse outcomes.

**Figure 6:**
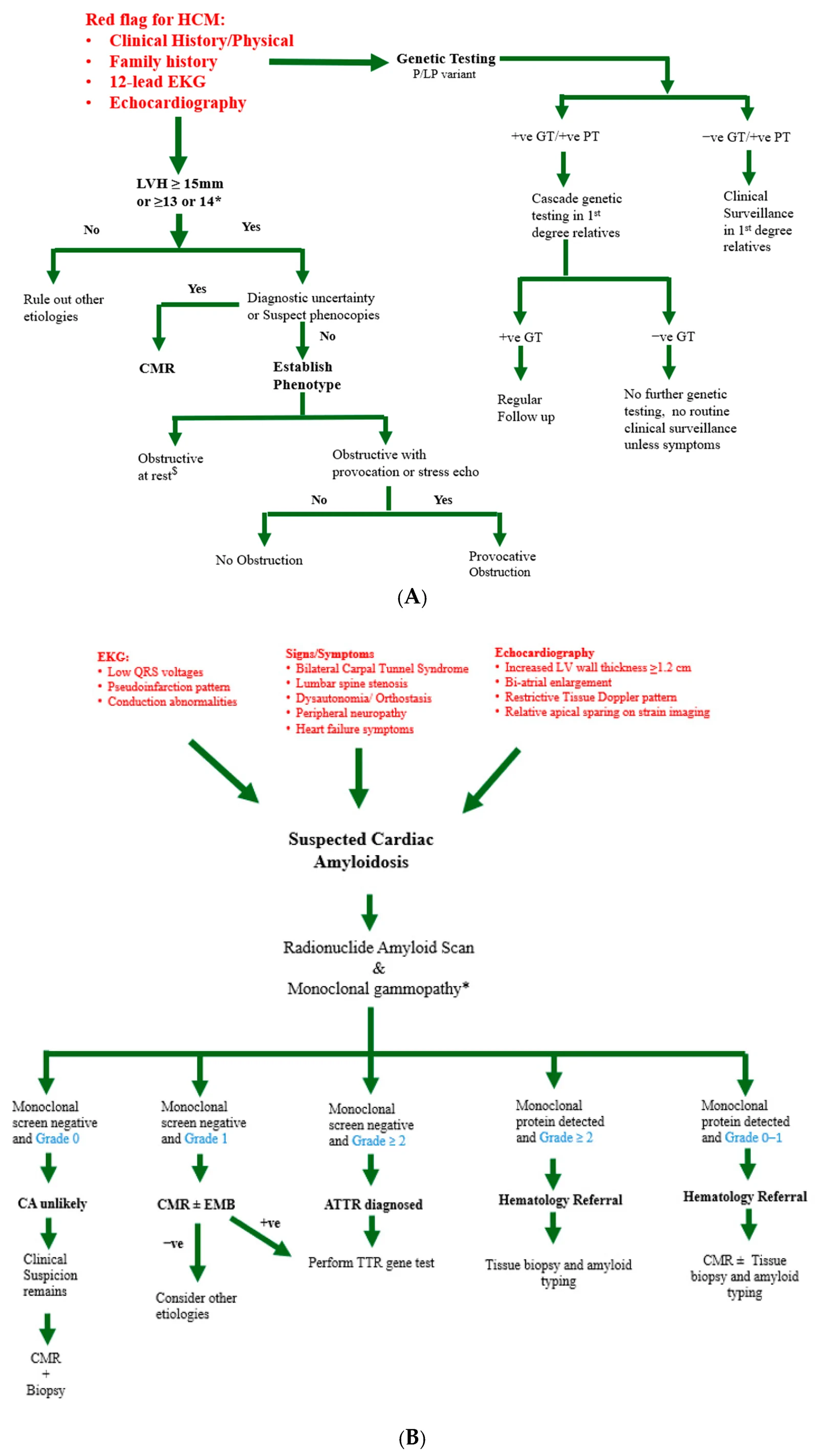
(**A**): Diagnostic algorithm of suspected hypertrophic cardiomyopathy. HCM: hypertrophic cardiomyopathy, LVH: left ventricular hypertrophy, CMR: cardiac magnetic resonance imaging, P/LP: pathogenic/likely pathogenic, GT: genotype, PT: phenotype. * With family history of HCM or SCD (sudden cardiac death) or positive genetic testing. ^$^ Defined as LVOT gradient of ≥30 mmHg. (**B**) Diagnostic algorithm of suspected cardiac amyloidosis. CA: cardiac amyloidosis, CMR: cardiac magnetic resonance imaging, ATTR: transthyretin cardiac amyloidosis, AL: light chain amyloidosis. * Serum/urine immunofixation and serum free light chain assay.

T2 mapping detects myocardial edema, confirming T2-weighted hyperintensity in HCM patients with ongoing injury or ischemia [72] (Table 2) (Figure 6A).

### 6.2. Cardiac Amyloidosis

CMR imaging offers advantages over echocardiography for the noninvasive evaluation of CA, owing to its high spatial resolution and superior capacity for myocardial tissue characterization [73,74]. Key CMR techniques include late gadolinium enhancement (LGE), T1 and T2 mapping, and extracellular volume (ECV) quantification. LGE primarily reflects expansion of the extracellular space and altered gadolinium kinetics resulting from myocardial amyloid deposition. Unlike ischemic heart disease, LGE in CA typically appears in a non-coronary artery distribution as seen in HCM, however, with different patterns compared to HCM that may be subendocardial, diffuse, focal, or transmural. Notably, the subendocardial pattern is characteristic of CA, while transmural involvement has been associated with worse outcomes [75,76] (Figure 5).

T1 mapping allows for early detection of myocardial infiltration, often preceding overt wall thickening. Elevated native (pre-contrast) T1 values not only aid in diagnosis but also serve as prognostic markers of disease severity [77,78]. By measuring pre- and post-contrast T1 times, CMR assesses extracellular matrix expansion, enabling differentiation between CA and other fibrotic cardiomyopathies. This technique also provides a quantitative measure for disease burden and treatment monitoring [73,77]. In addition, T2 mapping plays a complementary role by detecting myocardial edema, further enhancing risk stratification [79] (Table 2) (Figure 6B).

## 7. Nuclear Tracer Scan

Technetium-labeled nuclear scintigraphy, particularly with ^99mTc-pyrophosphate (PYP), has become a cornerstone in the noninvasive diagnosis of ATTR [80,81]. When used alongside serum immunofixation electrophoresis and serum free light chain (FLC) assays to exclude monoclonal gammopathy, Tc-PYP imaging demonstrates excellent diagnostic performance with high sensitivity and specificity for ATTR [82]. This approach has significantly reduced the need for tissue biopsy in most cases of ATTR, although histological confirmation remains necessary for diagnosing AL [82].

Importantly, the noninvasive diagnostic pathway for ATTR requires systematic exclusion of monoclonal gammopathy using serum free light chains together with serum and urine immunofixation. In the absence of monoclonal protein, Grade ≥ 2 uptake on bone-tracer scintigraphy, with confirmation of true myocardial uptake by SPECT or SPECT/CT, supports a non-biopsy diagnosis of ATTR. Conversely, when monoclonal studies are abnormal, scintigraphy uptake alone cannot reliably distinguish ATTR from AL, and further evaluation, often including histological confirmation with amyloid typing, is required [83].

**Figure 7:**
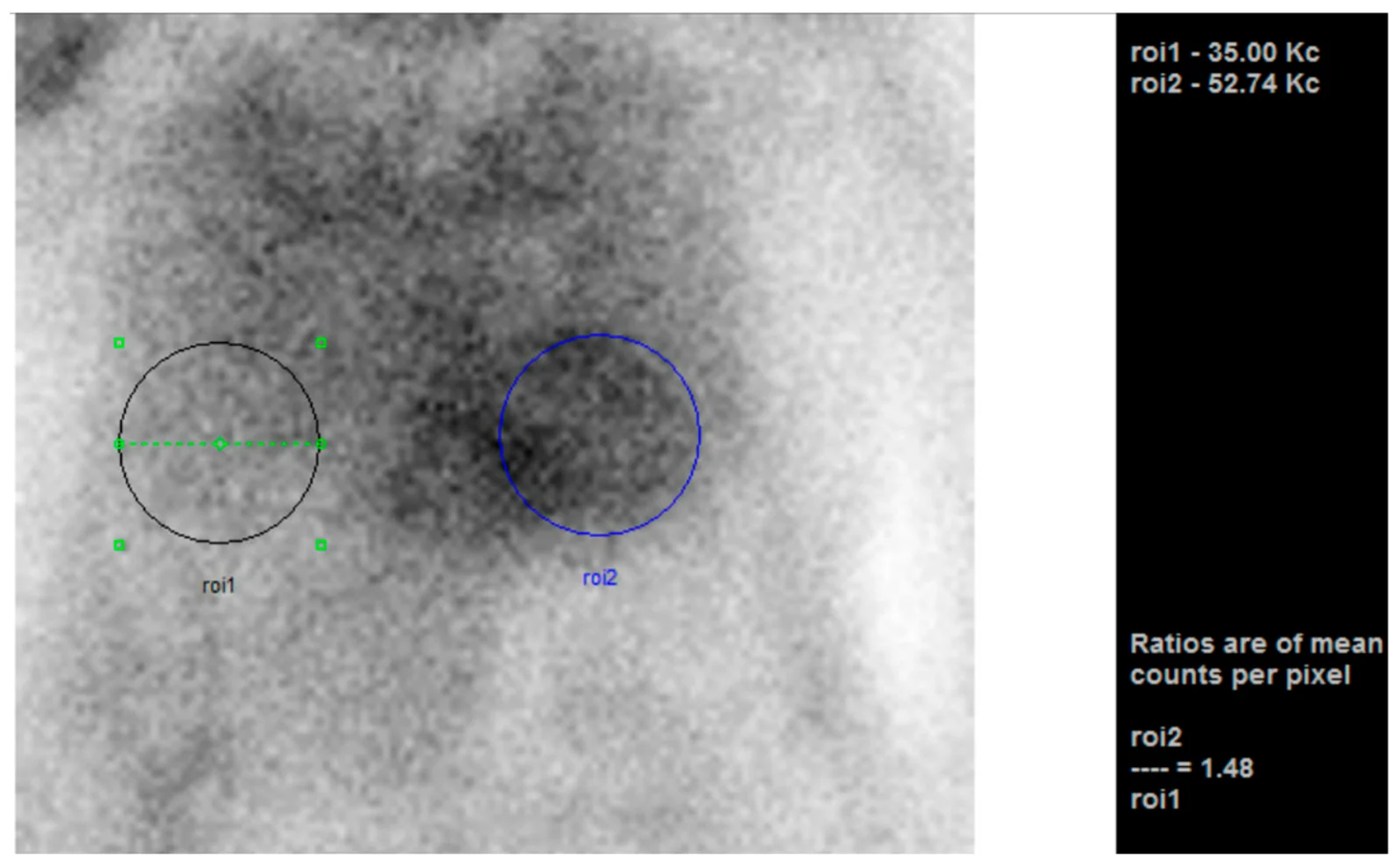
Nuclear 99mTechnetium-Pyrophosphate scan shows Perugini score of Grade 3, myocardial uptake greater than rib uptake with absent-to-no bone uptake, heart-to-contralateral ratio 1.5.

The diagnostic principle of Tc-PYP scintigraphy lies in the selective uptake of the radiotracer by ATTR deposits. Myocardial uptake is visually graded in comparison to rib (bone) uptake: Grade 2 (uptake equal to bone) and Grade 3 (uptake greater than bone) are considered highly specific for ATTR in the absence of a monoclonal protein [84]. Additionally, the heart-to-contralateral (H/CL) ratio serves as a semi-quantitative marker that can enhance diagnostic accuracy, though discrepancies may occur between planar imaging and calculated H/CL ratios [81] (Figure 7).

To confirm true myocardial tracer localization and distinguish it from blood pool activity, single-photon emission computed tomography is routinely employed. This step is critical for accurate interpretation and avoids false positives due to extracardiac signal. Overall, Tc-PYP scintigraphy—when interpreted in the appropriate clinical context—offers a noninvasive, highly effective method for diagnosing ATTR and differentiating it from other forms of cardiac amyloidosis [82].

## 8. Knowledge Gaps and Future Directions

Despite advances in genetic and molecular diagnostics, important knowledge gaps remain in HCM and CA. In HCM, genetic testing can identify pathogenic or likely pathogenic sarcomeric variants and facilitate family screening, although a causative variant is not identified in a substantial proportion of clinically diagnosed patients. In contrast, genetic testing has a different role in CA. AL amyloidosis is an acquired plasma cell disorder and is not diagnosed by germline genetic testing, while ATTRwt occurs in the absence of a pathogenic germline TTR variant. Once ATTR is established, TTR gene sequencing is performed to distinguish hereditary ATTRv from ATTRwt, with important implications for genetic counseling and family screening [83]. Future studies should continue to refine genotype–phenotype relationships in HCM and ATTRv and clarify the clinical significance of pathogenic variants across diverse populations.

The therapeutic landscape for HCM and CA has evolved with the emergence of targeted therapies. While myosin inhibitors such as mavacamten and aficamten have shown improvement in functional capacity, structural, and biomarker parameters in obstructive HCM [85,86], their long-term outcomes, role in early disease, and application in non-obstructive HCM require further studies. In ATTR, while tafamidis has demonstrated favorable outcomes in short-term studies [87], its high cost remains a barrier to widespread access, with cost-effectiveness analyses suggesting that substantial price reductions are needed to meet accepted thresholds. More recently, acoramidis, another TTR stabilizer, has demonstrated clinical efficacy and expanded the therapeutic options for ATTR [88]. In addition, vutrisiran, an RNA interference therapeutic that reduces hepatic TTR production, demonstrated reductions in all-cause mortality and recurrent cardiovascular events and preserved functional capacity and quality of life in the HELIOS-B trial [89], and is now approved for the treatment of ATTR. The availability of TTR stabilizers and gene-silencing therapy represents an important expansion of disease-modifying treatment for ATTR; however, comparative effectiveness, optimal patient selection, sequencing or combination of these therapies, and their long-term effects on survival and quality of life require further investigation. Future studies should prioritize long-term and real-world comparative data to better define the optimal use of these therapies.

Prognostic markers in HCM and CA have advanced, yet significant knowledge gaps persist, prompting focused future research. In HCM, contemporary risk models, such as the HCM Risk-SCD calculator, may not adequately predict SCD in certain subgroups, particularly those with apical HCM. Developing phenotype-specific risk models could enhance predictive accuracy. In CA, the staging systems have been devised that can accurately predict prognosis in ATTRwt and AL. However, their utility in individual ATTRv genotypes has not been studied. The phenotypic variability in ATTRv makes it challenging to devise a prognostic tool that is widely applicable to ATTRv population. Future studies are needed that could integrate genetic testing into prognostic models, which may have the potential to inform personalized treatment strategies.

## 9. Conclusions

Although HCM and CA are pathophysiologically distinct entities, they share overlapping clinical and imaging features—most notably increased myocardial wall thickness—which contributes to diagnostic ambiguity, especially in the absence of overt systemic manifestations. Despite advances in non-invasive imaging, underdiagnosis remains a common issue. The integration of advanced imaging modalities, genomic profiling, and emerging therapeutics offers a pathway toward earlier detection, individualized management, and improved outcomes. Future efforts should prioritize multicenter studies, inclusive registries, and broader implementation of risk stratification tools to address persistent diagnostic and therapeutic gaps in these complex cardiomyopathies.

## Figures and Tables

**Table 2:** Comparison of CMR characteristics in HCM versus CA.

Feature	HCM	CA
**Morphological Abnormalities**	Abnormal papillary muscle insertion and hypertrophy MV attachment variations	RV free wall and atrial septal thickening
**LGE**	Patchy, focal, mid-wall LGE at sites of maximal hypertrophy Non-coronary distribution LGE extent correlates with SCD risk	LGE in subendocardial, transmural, or diffuse pattern Non-coronary distribution Transmural LGE linked to worse prognosis
**T1 Mapping**	Elevated native T1 correlates with disease severity, wall thickness, and fibrosis	Elevated native T1 identifies early amyloid deposition, can precede LVH; diagnostic and prognostic relevance
**ECV**	Differentiates HCM from athlete’s heart Correlates with fibrosis burden and outcomes	Very high ECV reflects interstitial expansion, helps distinguish CA from other cardiomyopathies Tracks therapy response
**T2 Mapping**	Detects myocardial edema, useful in ischemic injury evaluation and prognostication	Identifies active disease progression, helps characterize tissue along with T1 mapping and LGE

HCM: hypertrophic cardiomyopathy, CA: cardiac amyloidosis, LV: left ventricle, MV: mitral valve, RV: right ventricle, SCD: sudden cardiac death, LGE: late gadolinium enhancement, LVH: left ventricular hypertrophy, ECV: extracellular volume.

## Data Availability

No new data were created or analyzed in this study. Data sharing is not applicable to this article.
